# Dupilumab and Blood Eosinophilia: A Disease‐Specific Phenomenon?

**DOI:** 10.1111/all.16610

**Published:** 2025-06-10

**Authors:** Andrea Portacci, Remo Poto, Gilda Varricchi, Giovanna Elisiana Carpagnano

**Affiliations:** ^1^ Institute of Respiratory Disease, Department of Translational Biomedicine and Neuroscience University “Aldo Moro” Bari Italy; ^2^ Department of Translational Medical Sciences and Center for Basic and Clinical Immunology Research (CISI) University of Naples Federico II, World Allergy Organization (WAO) Center of Excellence Naples Italy; ^3^ Istituti Clinici Scientifici Maugeri‐IRCCS Scientific Institute of Telese Terme Benevento Italy; ^4^ Institute of Experimental Endocrinology and Oncology (IEOS), National Research Council Naples Italy

**Keywords:** asthma, atopic dermatitis, COPD, CRSwNP, Dupilumab, eosinophilic esophagitis, hypereosinophilia

**Abbreviations**
AD: atopic dermatitis
COPD: chronic obstructive pulmonary disease
CRSwNP: chronic rhinosinusitis with nasal polyposis
EoE: eosinophilic esophagitis
IL‐4Rα: interleukin (IL)‐4 receptor alpha subunit
ILC2: group 2 innate lymphoid cells
INF‐γ: interferon γ
OCS: oral corticosteroids
ST2: receptor suppression of tumorigenicity 2
TNF‐α: tumor necrosis factor
VCAM‐1: vascular cell adhesion molecule 1

Dupilumab, a fully human monoclonal antibody that targets the interleukin (IL)‐4 receptor alpha subunit (IL‐4Rα) blocking IL‐4 and IL‐13 signaling, has revolutionized the treatment landscape for several type 2 (T2) inflammatory diseases, including severe asthma, chronic rhinosinusitis with nasal polyposis (CRSwNP), atopic dermatitis (AD), and eosinophilic esophagitis (EoE) [1]. Despite its therapeutic efficacy, the occurrence of transient blood eosinophilia during dupilumab treatment across different T2 diseases remains poorly understood. This phenomenon is generally not associated with clinical symptoms or impact on efficacy, occurs in the first few weeks, and returns to baseline or lower by the end of the treatment period. As reported by Wechsler et al. in a post hoc analysis of dupilumab trials involving patients with severe asthma, CRSwNP, and AD, transient blood eosinophilia occurs in a subset of individuals. This finding suggests that transient blood eosinophilia may be linked to a specific endotype of T2 inflammation that predisposes individuals to this response [2]. Nevertheless, while several molecular mechanisms have been proposed to explain the presence of blood eosinophilia during the blockage of the IL‐4/IL‐13 axis in patients with severe asthma, CRSwNP, and AD, the absence of this phenomenon in chronic obstructive pulmonary disease (COPD) and EoE remains elusive. This discrepancy raises key questions about whether blood eosinophilia is primarily driven by dupilumab mechanisms of action or by underlying disease‐specific immune pathways. Moreover, understanding these differences could have practical implications for clinical management, particularly in terms of monitoring adverse effects and tailoring treatment strategies.

The rise in blood eosinophils observed during dupilumab treatment has been attributed to multiple mechanisms, highlighting the role of cytokine dynamics and eosinophil trafficking. One hypothesis suggests that dupilumab‐induced eosinophilia results from altered vascular cell adhesion molecule‐1 (VCAM‐1) expression, a key molecule mediating eosinophil adhesion to endothelial cells. Blocking IL‐4/IL‐13 reduces VCAM‐1 expression, leading to decreased eosinophil migration from circulation into tissues, thereby increasing eosinophil counts in the blood [3, 4]. However, the expression of VCAM‐1 differs across diseases. Patients with COPD could exhibit a higher expression of VCAM‐1, especially those with increased cardiovascular risk [5]. This augmented VCAM‐1 expression could facilitate eosinophil retention within tissues, thereby preventing their accumulation in the bloodstream despite IL‐4/IL‐13 axis blockade (Figure 1). Similarly, in patients with EoE, VCAM‐1 is highly expressed on the esophageal vascular endothelium, eosinophils, and mast cells [6]. In EoE, VCAM‐1 expression is primarily driven by IL‐18, a cytokine involved in some aspects of both T2 and non‐T2 inflammation [7]. This increased expression may facilitate eosinophil tissue retention, explaining the lack of blood eosinophilia in EoE patients treated with dupilumab. From this perspective, future experimental models should directly assess the relationship between IL‐4/IL‐13 suppression and VCAM‐1 levels in serum and tissue among patients with T2 severe asthma, eosinophilic COPD, and EoE, to clarify the hypothesized role of this mechanism in the pathogenesis of blood eosinophilia following dupilumab administration. However, given that VCAM‐1 expression can be influenced by multiple cytokines (e.g., IL‐1, tumor necrosis factor (TNF)‐α, and IL‐18) [6, 8], it would be advisable to employ both in vivo and in vitro models to comprehensively address the potential confounding effects of alternative inflammatory pathways. This approach would facilitate a more precise definition of the specific role of IL‐4/IL‐13 axis suppression in modulating VCAM‐1 expression and regulating eosinophil migration across these distinct disease settings.

**FIGURE 1:**
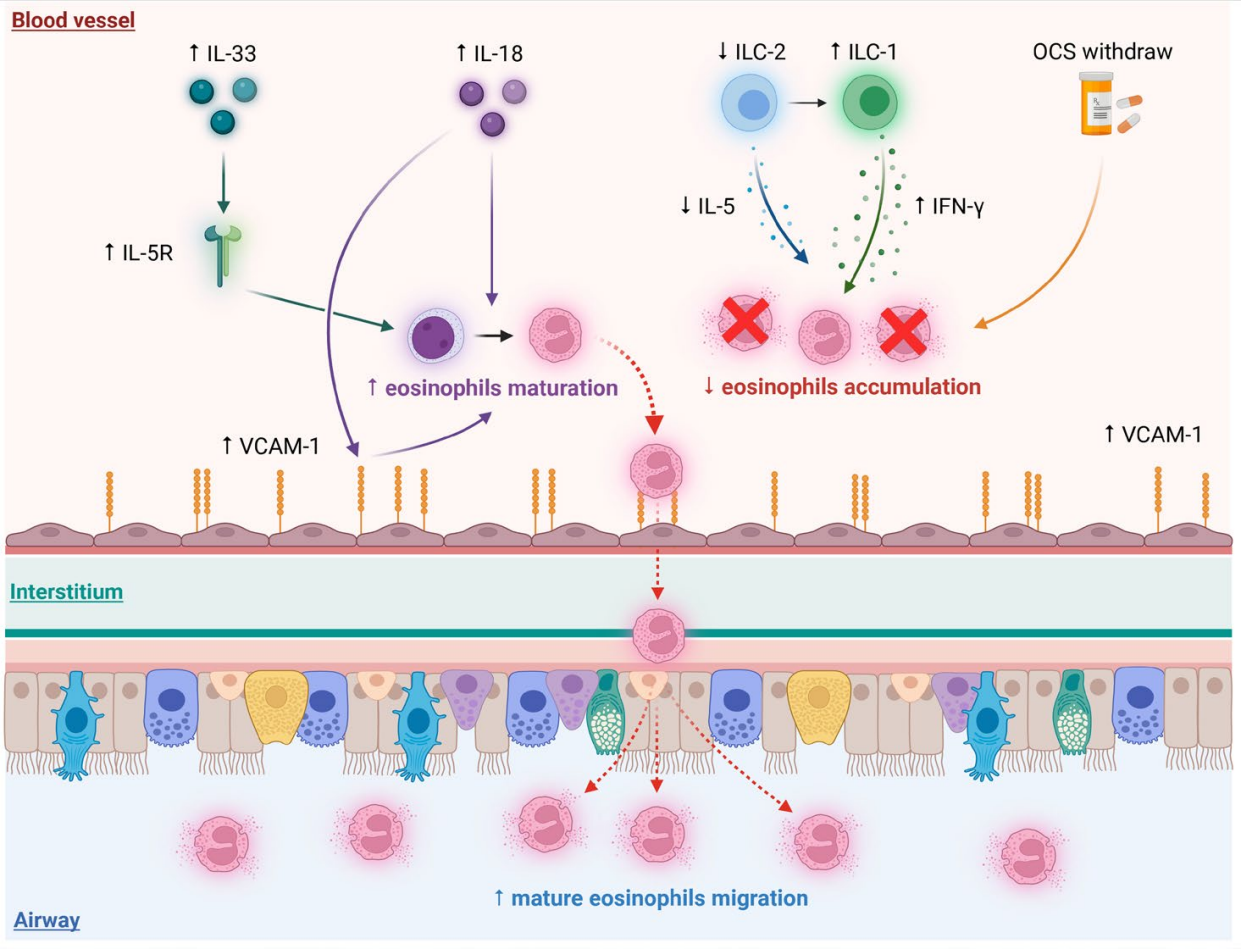
Possible mechanisms explaining the absence of transient blood eosinophilia in patients with COPD and EoE during dupilumab treatment. IL‐33 and IL‐18 could empower eosinophils maturation and migration from blood vessels to the airways. IL‐18 can also induce vascular cell adhesion molecule 1 (VCAM‐1) expression and group 2 innate lymphoid cells (ILC2) shift towards ILC1, reducing eosinophils bloodstream accumulation. Figure created using Biorender (https://biorender.com/).

Another proposed mechanism involves the differential expression of IL‐18 across different T2 diseases. IL‐18 appears to play a central role in various pathophysiologic mechanisms of COPD and EoE. IL‐18 is overexpressed in patients with COPD, asthma [9] and EoE [6, 8] compared to healthy individuals, orchestrating both T1 and T2 inflammation [7]. In mouse models, this cytokine promotes naïve eosinophil migration and differentiation into their inflammatory subtype (CD101^+^CD274^+^) with an IL‐5 independent mechanism, potentially bypassing the effects of IL‐4/IL‐13 inhibition on eosinophil trafficking [10]. Furthermore, IL‐18 has been shown to enhance the expression of VCAM‐1, further facilitating eosinophil tissue retention [6]. Since the IL‐4/IL‐13 axis induces the migration and maturation of cord and peripheral blood eosinophil progenitors (CD34^+^ CD45^+^) but does not influence the migration of committed or mature cells [11], IL‐18 activity on eosinophils maturation could provide a possible mechanism linking COPD and EoE to the absence of blood eosinophilia during dupilumab treatment. However, this mechanism remains speculative, as human data on IL‐18‐driven, IL‐5‐independent eosinophil migration in COPD and EoE are lacking. Furthermore, given the elevated IL‐18 levels in asthma as well, future research should investigate whether IL‐18 promotes eosinophil differentiation and migration through distinct pathways in asthma versus COPD and EoE.

A similar mechanism may involve IL‐33, an alarmin with pleiotropic effects on immune response in T2 inflammatory diseases [12]. IL‐33 promotes the expansion of eosinophil precursors and the upregulation of IL‐5R, inducing eosinophil commitment and maturation via IL‐5 [13]. In patients with EoE, IL‐33 is overexpressed on the esophageal epithelium [14] while its receptor suppression of tumorigenicity 2 (ST2) is frequently exposed on the surface of esophageal‐infiltrating eosinophils [15]. In the context of COPD, both IL‐33 and ST2 are overexpressed, especially in former smokers [16] and in response to a specific stimulus like lipopolysaccharide [17]. However, the absence of studies directly comparing sputum IL‐33 levels in patients with COPD vs. asthma prevents a definitive conclusion about this potential mechanism. As reported by Abdo et al., former smokers with COPD and a more severe GOLD stage exhibit the highest sputum IL‐33 expression, even when indirectly compared to patients with T2 severe asthma [18]. These findings suggest that the inflammatory environment in COPD and EoE fosters the presence of a greater proportion of committed or mature eosinophils, not dependent on IL‐4/IL‐13 for their migration to the target organ. This aspect could explain the absence of blood eosinophilia in COPD and EoE during dupilumab treatment, as the IL‐33/ST2 axis may bypass the effects of IL‐4/IL‐13 inhibition. Future investigations should focus on profiling IL‐33 on sputum and/or airway biopsies of patients with COPD and asthma, with an emphasis on distinguishing IL‐33 levels across different pheno‐endotypes.

Another potential mechanism explaining the increase in blood eosinophils observed during dupilumab treatment involves the reduction of IL‐13 levels, which may lead to NF‐kB upregulation and a subsequent increase in IL‐5 production [19]. This cytokine asset could result in the imbalance between eosinophil production in the bone marrow, driven by IL‐5 hyperproduction, and their reduced tissue migration due to IL‐4/IL‐13 blockage. However, in patients with EoE and COPD, IL‐18 and IL‐33 may act as alternative drivers of eosinophil commitment and migration even in the context of increased IL‐5 levels. Moreover, data comparing IL‐5 levels in patients with T2 severe asthma and eosinophilic COPD are lacking; hence, it can be speculated that patients with COPD could have a lower baseline IL‐5 production compared to asthmatic patients. This hypothesis is further reinforced by recent evidence on the role of group 2 innate lymphoid cells (ILC2), which are key resident immune cells promoting T2 inflammation and IL‐5 production [20]. While ILC2 are overexpressed in EoE [21], they are usually reduced in patients with COPD [22]. Moreover, IL‐18 can induce, along with IL‐12, the conversion of ILC2 towards ILC1, characterized by reduced production of IL‐5 and IL‐13 and increased interferon (IFN)‐γ secretion. From this perspective, studies on a comprehensive research model linking IL‐5 levels, ILC2 to ILC1 shift, and the role of IL‐18/IL‐33 production in patients with COPD could provide a more robust support for the hypothesis of a reduced IL‐5‐dependent stimulus on eosinophil entrapment after IL‐4/IL‐13 inhibition [22].

Finally, some authors have questioned the tapering of oral corticosteroids (OCS) as a potential contributor to blood eosinophilia observed during IL‐4/IL‐13 treatment. OCS discontinuation after a positive clinical and functional response to dupilumab could have removed steroid‐mediated suppression of eosinophil production and trafficking, leading to blood eosinophilia. This hypothesis is supported by data from the VENTURE trial, where approximately half of the asthmatic patients discontinued OCS use after 24 weeks of dupilumab treatment [23]. In contrast, no OCS use was reported in COPD NOTUS and BOREAS studies [24], while systemic glucocorticoids were explicitly prohibited for at least 3 months prior to baseline in the EoE trial [25]. These differences highlight the potential role of corticosteroid withdrawal in the emergence of blood eosinophilia.

The phenomenon of dupilumab‐induced blood eosinophilia highlights the complex interplay between drug mechanisms and disease‐specific pathophysiology. While eosinophilia associated with dupilumab is prominent in asthma, CRSwNP, and AD, its absence in COPD and EoE patients underscores the need for targeted experimental studies to validate the hypothesized involvement of alternative inflammatory pathways, such as IL‐18 and IL‐33 signaling, as well as the role of ILC2 plasticity.

Given these differences, clinicians should consider disease‐specific monitoring strategies. In particular, the absence of transient blood eosinophilia in COPD and EoE suggests that routine eosinophil counts may have limited utility in these patients when assessing dupilumab treatment response. A deeper understanding of these mechanisms will enhance therapeutic outcomes and safety in patients receiving dupilumab.

## Author Contributions

**Andrea Portacci:** supervision, conceptualization, investigation, writing – original draft, writing – review and editing. **Remo Poto:** supervision, conceptualization, investigation, writing – original draft, writing – review and editing. **Gilda Varricchi:** conceptualization, investigation, writing – original draft, writing – review and editing. **Giovanna Elisiana Carpagnano:** conceptualization, investigation, writing – original draft, writing – review and editing.

## Conflicts of Interest

Andrea Portacci reported payment or honoraria for lectures, presentations, speakers' bureaus, manuscript writing, or educational events from Astrazeneca, GlaxoSmithKline, Chiesi, Sanofi. Remo Poto reported personal fees from Astrazeneca and GSK. Gilda Varricchi reported research support from Astrazeneca. Giovanna Elisiana Carpagnano reported grants or contracts from Astrazeneca, Chiesi, GlaxoSmithKline, Sanofi, Grifols; payment or honoraria for lectures, presentations, speakers, bureaus, manuscript writing, or educational events from Astrazeneca, GlaxoSmithKline, Sanofi; support for attending meetings and/or travel from Astrazeneca, Menarini, Chiesi.

## Data Availability

The authors have nothing to report.
